# Short-Term Effects of Lower-Extremity Heavy Resistance versus High-Impact Plyometric Training on Neuromuscular Functional Performance of Professional Soccer Players

**DOI:** 10.3390/sports11100193

**Published:** 2023-10-04

**Authors:** Michał Boraczyński, José Magalhães, Jacek J. Nowakowski, James J. Laskin

**Affiliations:** 1 Faculty of Health Sciences, Collegium Medicum, University of Warmia and Mazury in Olsztyn, 11-041 Olsztyn, Poland; 2 Laboratory of Metabolism and Exercise (LaMetEx), Research Centre in Physical Activity, Health and Leisure (CIAFEL), Faculty of Sport, University of Porto, 4200-450 Porto, Portugal; jmaga@fade.up.pt; 3 Department of Ecology and Environmental Protection, University of Warmia and Mazury in Olsztyn, 10-719 Olsztyn, Poland; jacek.nowakowski@uwm.edu.pl; 4 School of Physical Therapy and Rehabilitation Sciences, University of Montana, Missoula, MT 59812, USA; james.laskin@mso.umt.edu

**Keywords:** maximal strength, short-distance sprinting, soccer training, vertical jumping performance

## Abstract

Background: To compare the effects of short-term 8 week heavy-resistance or plyometric training protocols (HRT or PLY) incorporated into regular soccer practice on measures of neuromuscular functional performance in professional soccer players, a single-blind randomized controlled trial was conducted. Methods: Forty-seven participants aged 22.3 ± 3.52 years were assigned to three groups: HRT (*n* = 15), PLY (*n* = 15), and control (CON; *n* = 17). The HRT group performed 3 sets and 10 repetitions twice a week using 80% of their baseline 1-RM (weeks 1–3), followed by 8 repetitions at 85% 1-RM (weeks 4–6), and 6 repetitions at 90% 1-RM (weeks 7–8) of 6 lower-body strength exercises with a 1 min rest period between sets. The PLY protocol involved a preparatory phase (weeks 1–2), followed by two 3-week progressive periods (weeks 3–5 and weeks 6–8). The plyometric sessions consisted of four jump exercises/drills with progressively increasing number of sets and total number of foot contacts. The rest intervals between repetitions and sets were 15 and 90 s, respectively. Outcome measures included tests assessing 10 and 30 m speed (t10m and t30m), one-repetition maximum half-back squat (1-RM squat), isokinetic peak torques for the quadriceps and hamstring muscles (Qcon and Hcon), countermovement jump (CMJ), and squat jump (SJ). Results: Two-way ANOVA detected main effects of time and group×time interactions for all examined variables, except t30m, 1-RM, and relative 1-RM. Post hoc analyses revealed significant increases in the HRT group (t10m: 6.3%, t30m: 7.1%; absolute 1-RM: 29.6%; relative 1-RM: 30.3%, Qcon: 24.5%; Hcon: 14.4%; CMJ: 5.9%; SJ: 7.2%, all *p* < 0.001) and the PLY group (t10m: 3.1%; t30m: 4.1%; absolute 1-RM:19.1%; relative 1-RM: 20.3%; Qcon: 12.6%; Hcon: 8.7%; CMJ: 3.3%; SJ: 3.5%, all *p* < 0.001). HRT was superior compared to PLY in relative 1-RM, Qcon and Hcon (all *p* < 0.001). In addition, we found knee muscular strength imbalance in 70.5% of participants from the total sample (H/Q ratio < 60%). The HRT and PLY protocols resulted in improved neuromuscular functional performance compared to the regular soccer regime. Conclusions: This study showed that during the pre-competitive season, additional HRT and PLY drills/exercises as a substitute for standard soccer training as part of a regular 90 min practice twice a week for 8 weeks, can produce acute physical performance-enhancing effects in professional soccer players.

## 1. Introduction

The comparative analyses of short-, medium-, and long-term effects of certain neuromuscular training methods (alone or combined) in the context of soccer-specific strength-based activities of professional athletes have been a key research topic in exercise science and physiology [1]. Addressing functional deficiencies and bilateral asymmetries of the lower extremities, as well as increasing or enhancing maximal strength, rate of force development (RFD), peak power output (PPO), speed, coordination, and muscle function in the stretch-shortening cycle (SSC) via traditional and/or alternative training methods is believed to be crucial for success of top-level soccer players [2,3].

Resistance training (RT) refers to specialized methods of physical conditioning and is considered a key strategy to improve neuromuscular function and subsequent on-field soccer performance [4,5]. There are multiple approaches to RT and a vast array of protocols and modalities commonly used by soccer coaches. The RT protocols often share several core principles: progressive overload, specificity, and the repeated bout effect, while RT modalities include free weights, machine weights, isokinetic devices, elastic bands, resisted running, and plyometrics [6].

Heavy-resistance-training (HRT) elicits a plethora of adaptations on a structural and neural level and HRT programs utilize relatively high load/high force (>70% one repetition maximum (1-RM)) and thus lower velocity, relatively few repetitions (4–8 repetitions), and isoinertial exercises like the bench press or squat [7]. However, although many specialists recommend the use in soccer players of HRT, resistive exercise with high loads (range 70–90% 1-RM), they also recommend high volumes, meaning sets of repetitions ending at or close to volitional fatigue [8,9]. From a traditional point of view, volitional fatigue or muscle failure is believed to provide adequate overload for optimizing maximal strength gains [10], but there is also evidence that this procedure can be counter-productive [11]. Specifically, it is believed that RT on leg extensor muscles primarily improves muscle power, kicking velocity, jumping ability, and sprint performance [12,13]. Such a training approach is meant to cause a maximal neural adaptation, specifically an increase in RFD and an improvement of intermuscular coordination, which will enhance functional explosive actions in soccer players such as single and repeated sprinting and/or jumps. Importantly, an adjusted periodized RT program could enhance the resting and/or exercise-induced neuromuscular responses to resistance exercise in professional soccer players [14,15]. However, a few studies reported marginally better improvements after combined power-band RT (4.15–6.35%) [16], or no improvements of vertical jump height after conventional RT methods [17,18]. These results indicate that the adaptive responses may be affected by a plethora of factors such as learning effect, expertise level, and exercise programming variables such as: intensity, volume, frequency, and recovery time between sets. Heavy-resistance training has been shown to elicit comparable effects on some lower-extremity performance variables as the plyometric (PLY) training mode [19]. However, explosive-type RT is considered to be a more effective training mode to improve vertical jump as compared to HRT [20]. 

The PLY training has been described as a sport-specific, effective, time-saving, and easy-to-implement training strategy in soccer players regardless of their age or competitive level [21,22]. This training method incorporates various types of body-weight jumping-type exercises such as countermovement jumps (CMJ), squat jumps (SJ), drop jumps (DJ), hopping, skipping, and alternate-leg bounding, which combine eccentric and concentric muscle actions based on SSC (rapid elongation of the muscle-tendon unit followed by an immediate shortening) [22]. Multiple variants of PLY training protocols have been reported for soccer including: without an external load, progressive or constant overload (e.g., ankle-loaded PLY) [23,24,25,26]. Regular lower-extremity PLY training including bilateral and/or unilateral jump exercises has consistently been shown to improve various measures and components of neuromuscular performance such as vertical and horizontal jumping ability [27,28,29], speed and acceleration [30], power and RFD [31], balance [32], agility [33], knee extension velocity during kicking the ball [34], and other soccer-specific high-intensity movements. However, these positive benefits of PLY training are not always found, there are a few studies that have reported no positive effects or even negative effects of PLY training, for example vertical jump height [35]. 

Several previous studies have been also inconclusive in determining the optimal design of PLY training (i.e., volume, intensity, frequency) to promote increased muscle strength and power and improve match performance (see the review by Ramirez-Campillo et al., [36]). PLY training has been shown to be able to induce a level of muscle activation (expressed as post-activation potentiation, PAP) comparable to that induced by HRT [37]. Moreover, both HRT and PLY have been used to improve jumping and sprint performance in elite soccer players [25] and combining of RT with jumping and running velocity exercises provides a more efficient method for improving activities that include acceleration, deceleration, and various jumps [38]. From a practical standpoint, the neuromuscular functional performance of soccer players is probably more significantly related to variables that are measured within the power-training load range (75–125% of body mass in half-squats) [39]. In this context, the optimal design and implementation of training strategies that enhance lower-extremity explosive performances in professional soccer players is still of significant interest to soccer coaches, strength and conditioning specialists, medical staff, and players. Unfortunately, given the inconsistent findings in the literature on the effects of HRT versus PLY, as well as significant heterogeneity in the study design and methodology (i.e., sample size, training modalities, age categories, primary and secondary outcome measures), further research is needed to clarify the acute effects of HRT and PLY interventions on specific performance adaptations in professional soccer players.

Therefore, the aim of this study is to compare the effects of the addition of an 8-week pre-season HRT or PLY training protocol, in male professional soccer players, on neuromuscular functional performance measured by: running velocity, 1-RM half-back squat strength, peak concentric isokinetic knee extensor and flexor torques, and vertical jump performance. We hypothesized that neuromuscular functional performance would adapt differently depending on the training protocol used during the 8-week intervention. Specifically, we tested the hypothesis that incorporating high-impact unilateral and bilateral power/PLY exercises into standard soccer training would induce more substantial improvement in vertical jump performance than HRT, while HRT would provoke better effects in sprint, 1-RM half-back-squat strength and isokinetic peak torques for the quadriceps and hamstring muscles.

## 2. Materials and Methods

### 2.1. Participants

Forty-seven professional male soccer players (values in mean ± SD; age: 22.3 ± 3.52 years, body height: 180.5 ± 5.43 cm, body mass: 74.2 ± 5.19 kg, body mass index: 22.8 ± 1.32 kg·m^2^, body fat: 12.6 ± 3.31%, fat-free mass: 64.4 ± 5.02 kg) were recruited to participate in this study. The participants were recruited from the first Polish professional soccer league (3 different soccer clubs) and had 14.2 ± 1.54 years of experience in structured soccer-specific training. Exclusion criteria included: a history of (i) unresolved musculoskeletal disorders; (ii) lower-extremity reconstructive surgery in the last 2 years, or (iii) taking performance-enhancement drugs including anabolic steroids. Additionally, the participants from HRT and PLY groups were asked to cease any additional lower-limb RT for the entirety of this study to minimize the confounding effects.

In this single-blind randomized controlled design, participants were randomized into 3 groups after being blocked by tactical position. The three groups consisted of the following: heavy-resistance training group (HRT, *n* = 15), plyometric training group (PLY, *n* = 15), and a control group (CG, *n* = 17). The randomization and blocking process resulted in the same number of goalkeepers (*n* = 2), defenders (*n* = 4), midfielders (*n* = 6), and forwards (*n* = 3) were present in the HRT and PLY groups, while 2 more forwards were in the CG group. Detailed subject characteristics are provided in Table 1.

The study was conducted according to the World Medical Association Declaration of Helsinki. All the participants gave written informed consent documents, and this research was fully approved by the institutional Ethics Committee for Human Experiments (No. 7/2019) and was performed in accordance with national standards in sport and exercise science research.

### 2.2. Procedures

**Figure 1:**
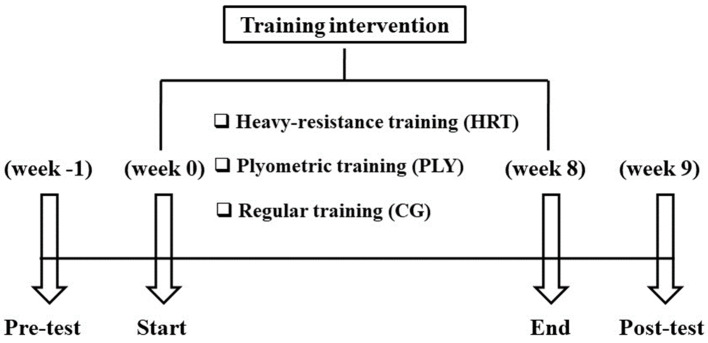
Timeline of the study procedures.

Pre- and post-tests were performed in the morning hours in a controlled laboratory setting (20.3–21.5 °C, 32–40% relative humidity, 749–761 mmHg) and sports hall. The participants were requested to maintain their diet and abstain prior to testing from caffeine and alcohol (minimum 24 h), avoid any strenuous exercise (minimum 48 h), and not use any non-prescription medication or supplements (minimum 48 h). All of the participants had experience in different modes of RT; all were accustomed to performing the 1-RM half-back squat, CMJ/SJ, and other resistive tests/exercises with correct technique, hence the potential learning effects were reduced. Nevertheless, prior to the testing session, the research manager provided participants with a full explanation of the study protocol, and participants were able to practice all tests in a familiarization session. The standardized warm-up period consisted of 5 min submaximal running at 9 km·h^−1^ followed by 5 min of practice jumps, dynamic stretching of the lower limbs (no static stretching exercises), half-squat with low loads (two sets of five repetitions at 50% of body mass with 2 min of rest between sets), and 2 submaximal sprints of 20 m. The testing sessions were divided into 2 days, in the following order—day 1: SJ, CMJ, peak concentric isokinetic knee extensor and flexor torques; day 2: 10 and 30 m linear sprints and 1-RM half-back squat. Tests were separated by 10 min rest intervals. Testing venue, time of the day and order of tests were identical during testing sessions for all groups. Days 1 and 2 were separated by 48 h. All tests were performed in a fatigue-free state. After the intervention period, a follow-up (post-test) was performed under the same conditions as the baseline testing (pre-test). Baseline data were collected during the 1 week prior to the 8-week intervention. The post-test was conducted after the participants competed a 7-day taper, i.e., total training volume was reduced by 35%, and sessions included low-intensity soccer training contents only to maximize their individual strength-power performance (see Figure 1). Only participants who attended at least 85% of the training sessions and completed both pre- and post-testing trials were included in data analysis.

#### 2.2.1. Anthropometry and Body Composition

Anthropometry and body composition analysis included measuring standing body height with a stadiometer (WB-150, Tryb-Wag ZPU, Zamość, Poland) and assessing body mass (BM), body mass index, percent body fat, and absolute fat-free mass with a MC-780U Multi Frequency Segmental Body Composition Analyzer (Tanita Corp., Tokyo, Japan) via bioelectric impedance analysis. All measurements were taken in the morning by a trained ISAK-accredited anthropometrist (Level 1) in compliance with the standard procedures recommended by the International Society for Advancement of Kinanthropometry.

#### 2.2.2. One-Repetition Maximum Test (1-RM)

Test with a single-repetition limit (1-RM) was performed in accordance with a standardized protocol proposed by Chelly et al. [40] that was also used effectively in our previous study [41]. The test was preceded by a supervised (Certified Strength and Conditioning Specialist) weight warm-up to ensure that exercises were performed correctly. The warm-up involved three sets of half-squats with progressive loads (40–70–85% of predicted 1-RM) with a decreasing number of repetitions (10–6–3). The half-back squat was performed from an upright position with the barbell set on the shoulders and firmly grasped with both hands. The participant then squatted to 90° of knee flexion and returned to the upright position with the legs fully extended. After two successful repetitions with a load approximately 5% below the participant predicted 1-RM an additional load of 1 kg was added and the procedure was repeated. If the second repetition could not be completed the test was terminated and the corresponding load was accepted as the individual’s 1-RM. A rest interval of 3 min was provided between the attempts. On average, 1-RM was achieved within 4–6 attempts. Absolute and relative values (normalized to BM) were used for analysis.

#### 2.2.3. Isokinetic Testing

Isokinetic peak torque for the knee flexors and extensors was performed on the dominant leg (designated as the preferred kicking leg). A standardized warm-up procedure including 5 min on a cycle ergometer and 5 min of static and dynamic stretches for the major lower limb muscle groups was completed prior to being positioned in a Biodex isokinetic dynamometer (BIODEX-3 Pro, Biodex Medical Systems Inc., Shirley, NY, USA). The dynamometer set-up was completed following the manufacturer’s guidelines. Restraints were applied to secure the chest, hips, thigh, and distal femur. The lateral femoral epicondyle was aligned with the axis of rotation of the dynamometer and the knee was fixed at 90° of flexion. All actions were performed through a range of 0–90° knee flexion and extension (with 0° being full knee extension). The participant performed a series of practice trials at 50%, 75%, and 100% perceived maximal effort before performing three 3 s maximal voluntary concentric quadriceps and hamstrings actions, at an isokinetic angular velocity of 1.05 rad·s^−1^ (60°·s^−1^), each separated by 30 s rest period. The researcher provided verbal motivation during each trial to ensure maximal effort. The peak torque outputs were calculated in absolute Newton-meters (Nm) and in Newton-meters relative to body mass (Nm·kg^−1^ BM). The best performance of the three repetitions was used in the data analysis. Based on concentric hamstring and quadriceps peak torque values (Hcon and Qcon), H/Q peak torque ratios were calculated for each participant.

#### 2.2.4. Vertical Jump Performance

Vertical jump performance was measured with two different jump types: (i) SJ and (ii) CMJ. Following an individual 5 min warm-up (practice jumps and jogging in place, dynamic stretching exercises) the participants began the SJ at a knee angle of 90°, avoiding any downward movement, and then performed an explosive vertical jump by pushing upwards, keeping their legs straight throughout. If a dipping movement of the hips was evident, then the trial was repeated. The CMJ was begun from an upright position, making a rapid downward movement to a 90° of knee flexion depth and simultaneously beginning to push-off. After each jump, the starting position was reset. An inter-repetition rest interval of 15 s was applied which is sufficient to fully recover vertical jumping ability and biomechanical-related parameters (e.g., applied force). Jump height (H_max_) was determined as the center of mass displacement, calculated from the recorded force and BM. One minute of rest was allowed between the 3 trials of each test and the best (highest) trial was used for analysis. All jumps were performed on a tensometric platform and analyzed using specialized MVJ v.3.4 software (JBA-Z, Staniak, Warsaw, Poland).

#### 2.2.5. Sprint Test

Running velocity was assessed on an indoor synthetic track and simultaneously tested for 10 m and 30 m sprint times. The test was preceded by a warm-up involving a low-intensity run (~10 km·h^−1^) for 5 min followed by 3 min of dynamic stretching exercises. The participants were instructed to begin the test from a stationary start position, with their preferred foot placed forward 0.3 m behind the starting line. After 1 practice trial, the participants completed two trials separated by an interval of at least 5 min of slow walking and rest. Sprint time was measured using infrared photoelectric cells (The Witty System, Microgate Srl, Bolzano, Italy). A photocell was placed at the start, at 10 m, and 30 m. The times for the 10 m and 30 m sprint (t10m and t30m, respectively) were measured with an accuracy of 0.001 s using. The fastest trial was used for analysis.

### 2.3. Training Interventions

The interventions were integrated into the players’ regular soccer training regime to conform to the constraints of the player’s schedules and were conducted during the pre-season conditioning period of the 2019/2020 competitive season.

Typical pre-season weekly training for the players included: soccer-specific training (5 sessions), physical conditioning (3–4 sessions), and competitive control match (1 game per week), totaling approx. 18 h per week on average. Sunday served as a day of rest. Routine single training session ~2 h comprised 15–20 min warm-up, ~20 min of technical drills; ~30 min of tactical drills; ~20 min of small-sided games; ~30 min of simulated competitive games; and 10 min of cool down. All groups followed the described routine training program for the study period. The participants from intervention groups completed the HRT and PLY specific drills/exercises as a substitute for standard soccer training (technical skills) within the scheduled 90 min practice sessions; two sessions per week over an 8-week period for a total of 16 sessions (Monday through Thursday). Previous studies have shown that this time period is long enough to elicit significant gains in physical performance in soccer players [23,42]. All training sessions were supervised by the same coach. The 72 h rest period between the HRT and PLY sessions was created to limit the potential effects of muscular fatigue on subsequent days. In addition, the players received a similar soccer-related load (i.e., session rating of perceived exertion, sRPE), and competitive training load (i.e., 8 competitive control matches during the 8-week intervention).

#### 2.3.1. Heavy-Resistance Training Protocol

The details of HRT program are displayed in Table 2. Heavy-resistance training, based on slightly modified HRT protocol proposed by Hermassi et al. [43], was progressive; loading levels were monitored continuously and adjusted to maximize muscle adaptation responses. The protocol emphasized a quick acceleration during the concentric phase, while the eccentric phase was executed in a controlled manner (approx. 3 s). For each exercise the players completed 3 sets (with 1 min rest period between sets), but the number of repetitions and training intensity (%1-RM) altered depending on the intervention period. Throughout the 8-week intervention the players completed during HRT session 3 sets of 6 lower-body strength exercises with 1 min rest period between sets. During the first 3 weeks players performed for each set 10 repetitions using 80% of their baseline 1-RM, then 8 repetitions at 85% 1-RM (weeks 4–6), and in the last two weeks 6 repetitions at 90% 1-RM (Table 2). Participants were supervised by the investigators and were allowed assistance on the last repetition.

#### 2.3.2. Plyometric Training Protocol

Specific details of the PLY training protocol are presented in Table 3. All PLY sessions were supervised and performed just after the warm-up for approx. 20 min to ensure that the players were prepared to gain optimal benefits from the incorporated training loads (calculated as volume × intensity). Training volume was determined by the number of ground contacts and training intensity was measured by the sRPE [44]. Exercise intensity was progressively increased from a level classified as medium to high and exercise volume was determined as high (i.e., total ground contacts). The PLY protocol involved the following periodization model: the first 2 weeks were a preparatory phase, followed by a 3-week progressive period (3–5 weeks) and final 3-week progressive period (6–8 weeks). Plyometric activity included multiple jumps (e.g., ankle hop, cone hop, skip hop, single- and double-leg vertical and lateral hurdle jump), horizontal and lateral bounding, power skipping, etc. Each plyometric session was composed of 4 different exercises/drills with progressively increased number of sets (i.e., 2 sets during the week 1, and 6 sets during the week 8) and total number of foot contacts. The rest interval between repetitions and sets was of approx. 5 and 90 s, respectively [45]. Previous studies had confirmed that these are optimal rest periods for PLY training. The participants were encouraged to obtain short contact time and maximal performance. The specificity of the applied protocol was that beside double-leg jumps, high-intensity single-leg exercises into both sagittal and lateral directions were also included.

### 2.4. Statistical Analysis

The basic model of analysis was the analysis of variance system (ANOVA) with repeated measures (R) and a fixed factor, which was the group of subjects (HRT, PLY, and CG). The general test of analysis of variance of variables in repeated measurements is based on the assumptions of using MANOVA and Lambda–Wilks test, where each F-test tests the multivariate effect of pre- and post-factor. The assumption of normality of the distribution of the sampled data and the distribution of the differences of the measurements were tested using the Shapiro–Wilk test. In the case of the Qcon variables, the analysis of variance was performed on the data after a logarithmic transformation, which brought the distributions in the samples to distributions that conformed to the normal distribution. The homoscedasticity of the variance in the case of comparisons of variables at the time of measurement pre and post, between groups of subjects was verified by Bartlett’s test, and since it was not possible to assess the homoscedasticity of the variance in the case of repeated measurements, the result of analysis of the normality of the distribution of measurement differences was used as the result eligible for parametric testing, or an adjusted F value according to the lower limit of the epsilon measure was used. Testing the significance of differences between pairs of compared variables was performed with the HSD-Tukey test for samples of different sizes or the Tamhane T2 test in the situation of unequal variances or the absence of a normal distribution of the differences of pre- and post-measurements. For the variables: t30m, 1-RM, and relative 1-RM, the comparison of means between research groups for pre-test and post-test results was based on the Kruskal–Wallis test and post hoc Dunn test, and the comparison over time (pre/post) in each group was based on the Wilcoxon test. For the variables: t10m, CMJ, SJ, Qcon and relative Qcon, partial eta square values (η^2^_p_) were used to determine the magnitude of the response to training protocols (effect size (ES) calculation with ∼0.01 = small effect, ∼0.06 = moderate effect, ≥0.14 = large effect). The *p* < 0.05 criterion was used for establishing statistical significance. The test–retest reliability (internal consistency) of performance assessments was determined using the Intra-Class Coefficients (ICCs) and Cronbach’s α. All statistical calculations were made using the STATISTICA™ v. 10.1 software package (StatSoft Inc., Tulsa, OK, USA) and IBM SPSS Statistics for Windows, version 26.0.0.1 (IBM Corp., Armonk, NY, USA).

## 3. Results

### 3.1. Testing Reliability

Test–retest reliability for the performance tests was strong with ICCs and Cronbach’s α ranging from 0.89 to 0.96 (ICC for the two-way mixed model is equal to Cronbach’s α). In addition, CVs ranged from 1.1% to 2.5% (Table 4).

### 3.2. Baseline Status

The initial level of athletic performance between the research groups was comparable, as there were no significant differences at baseline in any of the variables tested (*p* < 0.05).

### 3.3. Trial Adherence

The adherence to the 8-week training intervention was good; 87.2% of the prescribed training sessions were completed. The attendance rates for the research groups were as follows: HRT group = 86.8%, PLY group = 90.4%, and CG = 87.6%. Based on these results, all groups exceeded the predetermined attendance threshold (85%) across the intervention period. The reasons for participants’ absence from training sessions were illness and/or mild musculoskeletal strains (mainly involving the lower extremities). None of the players received a serious injury during the intervention period.

### 3.4. Training-Induced Effects on Neuromuscular Functional Performance

#### 3.4.1. Sprint Tests (10 m and 30 m)

**Figure 2:**
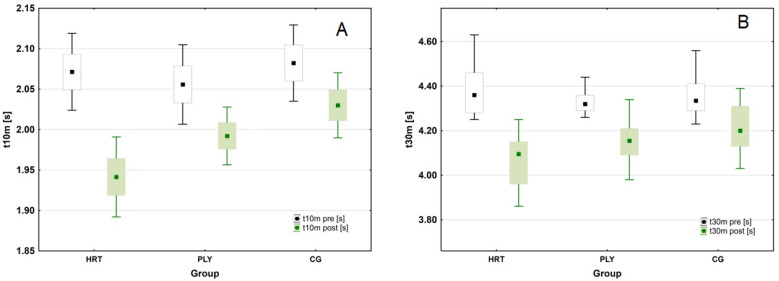
Changes in sprint scores following heavy-resistance training, plyometric training, and standard soccer regimen for 8-week intervention. (**A**) Changes in 10 m (t10m) sprint scores during the intervention period (box-and-whisker plots: t10m—the mean value is presented as a square, standard error of measurement (SEM) of the data are given by the box, and the whiskers indicate 95% confidence interval (CI)). (**B**) Changes in 30 m (t30m) sprint scores during the intervention period (t30m—the median is shown as a square, 25th to 75th percentiles (i.e., interquartile range, or IQR) of the data are given by the box, and whiskers indicate ± 1.5 IQR).

The ANOVA revealed a significant group × time interaction in sprint time at 10 m (t10m) (*F*_(2,43)_ = 16.162; *p* < 0.001; η^2^_p_ = 0.429; (large ES)). Post hoc analyses revealed the greatest pre- to post-training decreases in t10m scores in the HRT group (mean difference between test sessions: 0.132 s; ∆6.3%; Tukey test: *p* < 0.001), and smaller in the PLY and CG groups (mean difference between test sessions: 0.064 s; ∆3.1%; Tukey test: *p* < 0.001, and 0.052 s; ∆2.4%; Tukey test: *p* = < 0.001, respectively). At post-test the sprint time in the HRT group was significantly faster (by 0.09 s or ∆4.6%) than in the CG group (Tukey test: *p* = < 0.001). The sprint time at 30 m (t30m) in the total study sample was significantly slower at pre- compared to post-test (Wilcoxon test: Z = 5.905, *p* < 0.001, *n* = 47). The variation was significant in all groups (Wilcoxon test: HRT: Z = 3.296, *p* < 0.001, *n* = 15; PLY: Z = 3.296, *p* < 0.001, *n* = 15; CG: Z = 3.724, *p* < 0.001, *n* = 17). However, significant differences were present at the post-test (Kruskal–Wallis test: H_(2, n = 46)_ = 9.042, *p* = < 0.05), when the HRT group’s sprint time was significantly lower than the CG group’s (Dunn test: *p* = 0.008; by 0.14 s or ∆3.4%) (Figure 2).

#### 3.4.2. Vertical Jump Performances (CMJ and SJ)

**Figure 3:**
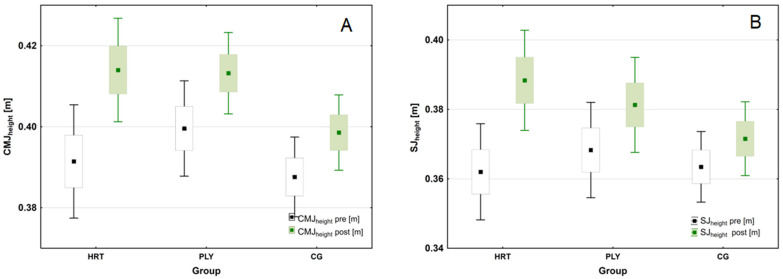
Changes in jump performance following heavy-resistance training, plyometric training, and standard soccer regimen for 8-week intervention. (**A**) Changes in countermovement jump heights (CMJ_height_) during the intervention period. (**B**) Changes in squat jump heights (SJ_height_) during the intervention period (box-and-whisker plots: for both variables—the mean value is presented as a square, standard error of measurement (SEM) of the data are given by the box, and the whiskers indicate 95% confidence interval (CI)).

A significant group × time interaction was also found in CMJ_height_ (*F*_(2,43)_ = 30.274; *p* < 0.001; η^2^_p_ = 0.585; (large ES)), and SJ_height_ (*F*_(2,43)_ = 113.791; *p* < 0.001; η^2^_p_ = 0.841; (large ES)), respectively. Further post hoc tests showed a similar and significant pre- to post-training increases in CMJ and SJ height performances in all research groups (CMJ_height_: HRT = 0.023 m, ∆5.9%; PLY = 0.013 m, ∆3.3%; CG = 0.011 m, ∆2.8%; SJ_height_: HRT = 0.026 m, ∆7.2%; PLY = 0.013 m, ∆3.5%; CG = 0.009 m, ∆2.5%; Tukey test: all *p* < 0.001), but with a greater within-group ES in HRT (1.037 and 0.923 for, respectively, CMJ_height_ and SJ_height_) when compared to PLY group (0.631 and 0.495 for, respectively, CMJ_height_ and SJ_height_) (Figure 3).

#### 3.4.3. The 1-RM Squat Performance (Absolute and Relative 1-RM)

**Figure 4:**
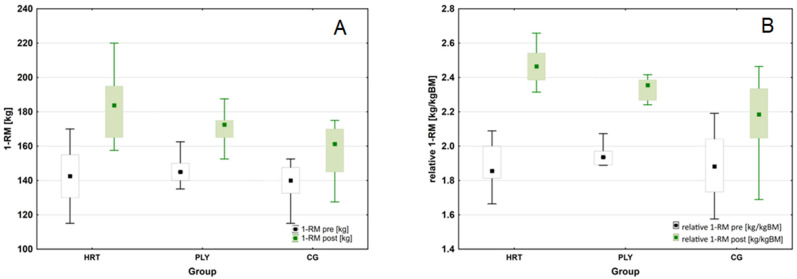
Changes in maximal strength performance following heavy-resistance training, plyometric training, and standard soccer regimen for 8-week intervention. (**A**) Changes in absolute one repetition maximum (1-RM) during the intervention period. (**B**) Changes in relative one repetition maximum (relative 1-RM) (box-and-whisker plots: for both variables—the median is shown as a square, 25th to 75th percentiles (i.e., interquartile range, or IQR) of the data are given by the box, and whiskers indicate ± 1.5 IQR).

We observed significantly higher levels of 1-RM at the post-test compared to the pre-test (Wilcoxon test: Z = 5.905, *p* < 0.001, *n* = 46). The variation was significant in all groups (Wilcoxon test: HRT: Z = 3.296, *p* < 0.001, *n* = 15; PLY: Z = 3.296, *p* < 0.001, *n* = 15; CG: Z = 3.724, *p* < 0.0002, *n* = 17). The average 1-RM showed significant differences between the groups at the post-test (Kruskal–Wallis test: H_(2, *n* = 46)_ = 14.833, *p* = 0.0006). In this time point, the 1-RM was significantly higher in the HRT compared to the CG group (by 24.9 kg or ∆15.7%; Dunn test: *p* = 0.0005). In addition, pre- to post-training increase in 1-RM in HRT group was substantially greater compared to PLY group (41.8 kg, ∆29.6% and 27.1 kg, ∆19.1%, respectively; Dunn test: *p* < 0.001). For the entire sample analyzed, there was a significantly higher level in relative 1-RM at the post- compared to the pre-test (Wilcoxon test: Z = 5.905, *p* < 0.001, *n* = 46), and the differences were significant in all groups (Wilcoxon test: HRT: Z = 3.296, *p* < 0.001, *n* = 15; PLY: Z = 3.296, *p* < 0.001, *n* = 15; CG: Z = 3.724, *p* < 0.001, *n* = 17). The average relative 1-RM variable did not differ between groups (Kruskal-Wallis test: H_(2, *n* = 46)_ = 0.452, *p* = 0.798), while it differed between groups significantly at the post-test (Kruskal–Wallis test: H_(2, *n* = 46)_ = 16.022, *p* = 0.0003). As before, the HRT group’s score was significantly higher than that obtained by the CG group (0.29 kg/kg·BM or ∆13.4%; Dunn test: *p* = 0.0002) (Figure 4).

#### 3.4.4. Peak Isokinetic Torque (Absolute and Relative Qcon and Hcon)

**Figure 5:**
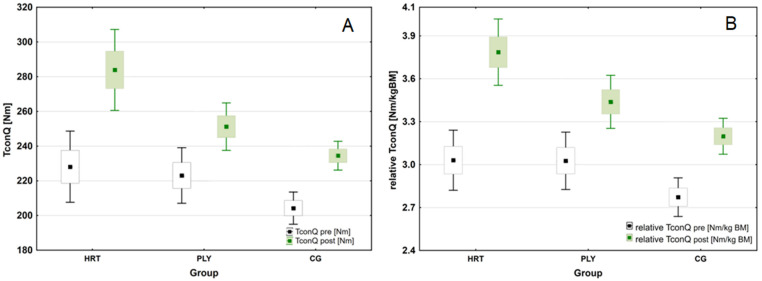
Changes in isokinetic strength following heavy-resistance training, plyometric training, and standard soccer regimen for 8-week intervention. (**A**) Changes in absolute peak concentric isokinetic knee extensor torque (Qcon) during the intervention period. (**B**) Changes in relative peak concentric isokinetic knee extensor torque (relative Qcon) during the intervention period (box-and-whisker plots: for both variables—the mean value is presented as a square, standard error of measurement (SEM) of the data are given by the box, and the whiskers indicate 95% confidence interval (CI)).

The analysis revealed a significant group × time interaction for peak concentric isokinetic knee extensor torque (Qcon) (*F*_(2,43)_ = 15.247; *p* < 0.001; η^2^_p_ = 0.415; (large ES)). Overall, the HRT group had a higher level of Qcon compared to the CG group (Tukey test: *p* = 0.003), Post hoc analyses also revealed significant increases in Qcon from pre-to post-training in the HRT (Δ24.5%, *p* < 0.001) and PLY (Δ12.6%, *p* < 0.01) groups without any significant changes in CG group (*p* > 0.05). Greater intra-group effect size was found for the HRT (ES = 0.424) when compared to either PLY or CG groups (ES = 0.152 and 0.074, respectively). At the post-test Qcon levels differed significantly between HRT and PLY groups (283.9 vs. 251.2 Nm; Tamhane test: *p* = 0.048); and between HRT and CG (283.9 vs. 234.5 Nm; Tamhane test: *p* = 0.002). Moreover, a significant group × time interaction was observed for relative Qcon (*F*_(2,43)_ = 25.061; *p* < 0.001; η^2^_p_ = 0.538; (large ES)). In general, the HRT group had a higher level of the variable compared to the CG group (Tukey test: *p* = 0.003), while it did not differ from the PLY group (Tukey test: *p* = 0.314). There was also noted an improved post-training relative Qcon in the HRT (*p* < 0.001) and PLY (*p* < 0.001) groups (Figure 5).

**Figure 6:**
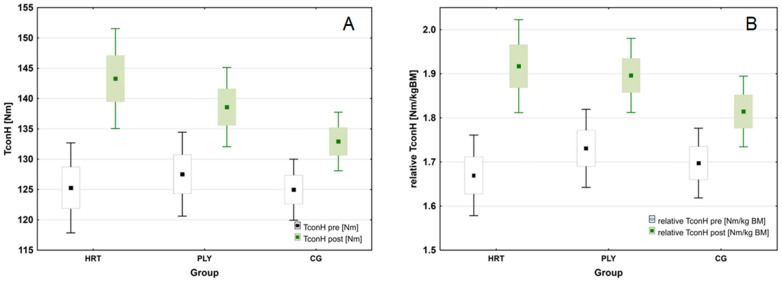
Changes in isokinetic strength following heavy-resistance training, plyometric training, and standard soccer regimen for 8-week intervention. (**A**) Changes in absolute peak concentric isokinetic knee flexor torque (Hcon) during the intervention period. (**B**) Changes in relative peak concentric isokinetic knee flexor torque (relative Hcon) during the intervention period (box-and-whisker plots: for both variables—the mean value is presented as a square, standard error of measurement (SEM) of the data are given by the box, and the whiskers indicate 95% confidence interval (CI)).

For the peak concentric isokinetic knee flexor torque (Hcon) a significant group × time interaction was noted (*F*_(2,43)_ = 72.966; *p* < 0.001; η^2^_p_ = 0.772; (large ES)). The post hoc tests revealed the greatest improvement from pre- to post-training in the HRT group (from 125.3 to 143.3 Nm, Δ14.4%, *p* < 0.01). Considering the peak torque values relative to body mass (relative Hcon), there was again a significant group × time interaction (*F*_(2,43)_ = 72.966; *p* < 0.001; η^2^_p_ = 0.772; (large ES)). Post hoc analyses also revealed significant increases for the HRT and PLY groups in relative Hcon (Δ15.1% and Δ9.8%, respectively, both *p* < 0.001) (Figure 6).

Regarding H/Q strength ratios for dominant knee there were no significant differences (*p* > 0.05) between PLY and CG groups for this measure at the pre-test (Δ57.1% and 61.2%, respectively), however CG group had significantly higher H/Q strength ratio (*p* < 0.05) compared to HRT group (Δ54.9%). In the post-test the significance level of between-group differences remained the same (CG vs. HRT, *p* < 0.05; HRT vs. PLY, *p* > 0.05; PLY vs. CG, *p* > 0.05). Interestingly, from pre- to post-test H/Q strength ratios decreased in all groups (mean % difference between test sessions: HRT = decrease by Δ4.4%, post-test: 50.5%; PLY = decrease by Δ1.9%, post-test: 55.2%; CG = decrease by Δ3.5%, post-test: 56.7%). The percentage of soccer players who presented knee imbalance, characterized by lower values than 60%, is depicted in Table 5.

## 4. Discussion

The present study utilized an 8-week single-blind randomized controlled study design to compare the effectiveness of the addition of two distinct training protocols, PLY, and HRT, to standard soccer pre-season training sessions on neuromuscular functional performance in professional soccer players.

In short, the results of this study show different and specific short-term selective training adaptations of both training protocols when integrated with regular soccer practice during the pre-season conditioning period. The outcomes also demonstrate that both HRT and PLY resulted in improved neuromuscular functional performance compared to the CG group. Specifically, the main findings of this study suggest that: (a) improvements in the sprint performance were comparable following the HRT and PLY protocols, so both protocols can be performed twice a week for 8 weeks without negatively affecting sprint performance; the 1-RM half-back squat performance of the HRT group was substantially better compared to the PLY group, but only in relative terms; percentage increases from pre- to post-training in the Qcon and Hcon were almost two-fold greater in the HRT group compared to the PLY group; the majority of soccer players (70.5%) presented knee muscular strength imbalances, expressed by an H/Q ratio of less than 60%; and surprisingly, the high-impact PLY protocol was not more effective in increasing vertical jump performance compared to HRT.

### 4.1. Sprint Performance

In several studies, 10 m and/or 30 m distances have been selected for sprint testing in soccer players [46,47]. In the present study, the analysis of the main effects for time in the sprint performance revealed significant and comparable pre- to post-training improvements following the HRT and PLY protocols. However, from the practical point of view, although the HRT group obtained better sprint scores at every time point, the percentage improvements were more meaningful following PLY compared to HRT protocol (t10m: 3.4% vs. 3.1%, and t30m: 4.2% vs. 2.4%, respectively). Nevertheless, the sprint improvements in HRT group, both t10m and t30m, were significantly better compared to CG group. These results are not surprising as previously a number of conditioning programs commonly implemented in professional soccer were shown to be effective in enhancing sprinting/running ability, including heavy-load squats [48], loaded CMJ activity [49], and either unloaded or loaded PLY exercises [50]. In soccer, marked improvement in sprinting is critically important in practical terms, because straight sprinting is the most frequent physical action in goal situations [51].

However, as hypothesized, we expected better improvement in sprint performance in the HRT group, not PLY. We based our assumption on a systematic review and meta-analysis by Seitz et al. [52] showing that increases in lower-body strength transfer positively to sprint performance. Indeed, multiple previous studies demonstrated strong correlation between 1-RM back squat performance with sprint performance [53,54,55]. For example, Styles et al. [56] reported strong correlations between the percentage change in relative 1-RM and 5, 10, and 20 m sprint times (*r* = 0.62, 0.78, 0.60, *p* < 0.001, respectively) and Comfort et al. [57] demonstrated similar changes of 5.9–7.6% in the sprint performance (5, 10, and 20 m) of elite rugby players after an 8-week RT program. Considering the velocity–strength relationship, Chaouachi et al. [58] demonstrated that 1-RM squat strength was the best single performance predictor for 5 and 10 m sprint times (*p* < 0.05). In contrast, Requena et al. [39] found that neuromuscular functional performance (especially 15 m sprint time) was poorly correlated with the isometric and isokinetic muscle strength measurements.

Previous observations suggest that HRT may differentially affect the initial acceleration phase (0–10 m) and the subsequent sprint interval (maximal-velocity phase, i.e., 10–30 m) [20]. Indeed, the heavy loading training procedure is oriented toward the early acceleration phase of the force–velocity (FV) spectrum [59]. Since 96% of sprints during a soccer game are shorter than 30 m, with 49% being <10 m [55], the improvements in the early acceleration phase are of greater practical importance. We suppose that the lack of marked sprint improvement in the HRT compared to the PLY group was likely due to the heavy loads used in the HRT protocol (80–90% 1-RM), which facilitate maximal voluntary contraction but, when used regularly, pose a risk of peripheral fatigue and concomitant declines in muscle function [60]. In addition, although participants from the HRT group trained the fastest motor units that produce the greatest force, it is possible that too few exercises involving movements with a rapid action limited their velocity-specific training response. Probably using lower loads on alternate training days would have resulted in greater improvements in the initial acceleration phase (10 m) of the sprint. However, we should remember that an improvement in one phase may be offset by a decline in another phase. On the other hand, the PLY protocol consisted of some sprint-specific PLY activities (e.g., hopping and bounding exercises), thereby increasing the degree of specificity of training exercises and inducing transference effect of added vertical/horizontal plyometrics on sprinting performance. In fact, several previous studies have demonstrated that PLY training-axis is decisive in determining neuromechanical training responses in high-level soccer players [61], and can effectively enhance sprint performance by improving SSC muscle function and consequently improving contractile performance [30,33,62]. In contrast, Herrero et al. [63] found no significant gains of SJ_height_, CMJ_height_, or 20 m sprint time with PLY training. Nevertheless, given the heterogeneous and multidimensional nature of sprint performance, perhaps the best option for improving linear and multidirectional sprint performance in the short term, in both youth and professional soccer players, is to adopt a combined RT and PLY program as part of the soccer regime [38,64].

### 4.2. Maximal Strength Performance

Since prerequisite levels of muscle strength are necessary to transfer velocity activities, it was formerly proven that the increases in 1-RM half-back squat values are responsible for running velocity improvements [53,55,56]. In this study, the soccer players from HRT group trained at 80–90% 1-RM, which can be considered a heavy training intensity [65] and can enhance the acute neuromuscular responses [66]. However, at the end of the intervention, absolute 1-RM values in half-back squat test were not significantly higher in the HRT group compared to the PLY group (i.e., 14.3 kg, 8.5%, *p* > 0.05), but in relative 1-RM there was a significantly higher score in favor of HRT (by 0.14 kg·kg^–1^ body mass (BM) or 13.4%, *p* < 0.05). Importantly, in the post-test 1-RM in the HRT group was 183.2 ± 18.9 kg and accounted the 244.6 ± 16.7% of their body mass (2.4 kg·kg^–1^ BM). Similarly, in the PLY group it was 168.9 ± 15.6 kg that represented the 230.7 ± 58.9% of their body mass (2.3 kg·kg^–1^ BM). The variation in this measure was comparable among the groups, and these short-term training responses could possibly induced transference effects, which in turn led to improved sprint times and vertical jump performance. However, these results were already expected for the HRT group, but such increments in 1-RM were actually surprising within the PLY group. Regarding the possible mechanisms, the positive changes in lower-extremity maximal strength might be related with improvements in neuromuscular function (e.g., increased neural drive to the agonist muscles, changes in the pattern of muscle activation) that are likely to occur in response to high-impact PLY training [22,67].

Moreover, the performance of both groups in absolute and relative 1-RM values in the half-back squat exercise was higher than the published data of Norwegian (171.7 ± 21.2 kg; 2.2 kg·kg^–1^ BM) [55], Polish (150.1 ± 15.1 kg; 2.0 kg·kg^–1^ BM) [41], Estonian (119.5 ± 26.2 kg; 1.7 kg·kg^–1^ BM) [39], and Greek (154.54 ± 15.7; 2.1 kg·kg^–1^ BM) [18] professional soccer players at the similar level of expertise. Considering the pre- to post-training gains in 1-RM half-back squat, other intervention studies (using four series of 4–5 repetitions) on professional soccer players reported gains, higher than in our study, from 115 to 176 kg (53%) and from 161 to 215 kg (33.5%) [68,69]. However, the results obtained in the current intervention are consistent with the study by Brito et al. [12], who showed improvements in this measure in the resistance, plyometric, and complex training groups by 22.8%, 17.3% and 24.2%, respectively (in our study, the percentage increases in the HRT, PLY, and CG groups were 29.6%, 19.1%, and 14.3%, respectively). Our results also corroborate with the study by Chelly et al. [40] who observed high 1-RM half-back squat gains in 17-year-old soccer players following RT program with heavy loads performed twice a week for 2 months (from 105 to 142 kg, 35%; the increase was only by 3.7% in the control group). To this end, the relative values of the 1-RM test recorded in this study almost reach the level of recommendation for professional soccer players, as Hoff and Helgerud [69] recommend a relative strength of 2.75-fold body mass in the half-back squat.

Isokinetic dynamometry is considered the gold standard for assessing muscle strength and imbalances between the knee flexors and extensors in soccer players [70]. Isokinetic variables related to muscle strength, such as isokinetic torque of the knee extensors and flexors and the conventional hamstring/quadriceps torque ratio (H/Q), are traditionally extracted from the isokinetic test. In this study, the percentage increases from pre- to post-training in the peak concentric isokinetic torques of knee extensors and flexors (Qcon and Hcon) were almost two-fold greater in the HRT group compared to the PLY group (Δ24.5%, versus Δ12.6%, and Δ14.4% versus Δ8.7%, respectively). These results show, in combination with the observed 1-RM gains, that HRT strongly increased the available muscle contraction force in the relevant muscle groups. However, a parallel negative aspect of this study was the relatively high percentage of players, in all groups (HRT = 80.0%, PLT = 66.7%, and CG = 64.7%), who presented a knee muscular strength imbalance (quadriceps vs. hamstrings), characterized by the H/Q strength ratio of less than 60% (alternative notation: H/Q ratio < 0.60).

With reference to the literature, the average values of the conventional H/Q strength ratio range from 57–66% [71,72,73], while H/Q ratios below 60% [74,75] have been utilized as cut-off values for identifying muscle strength imbalances in the knee joint. The H/Q strength ratio of less than 60%, as observed in majority of soccer players tested in this study, may increase the risk of hamstring strain injury more than four-fold, compared with players with normal H/Q ratios during the pre-season [76]. In the present study, relatively low but similar H/Q strength ratios were observed, as for example in de Lira et al. [77] study, who investigated thigh muscles isokinetic strength, H/Q strength ratios, and bilateral strength comparisons in athletes practicing different types of soccer (professional soccer, futsal, and beach soccer; their H/Q ratios were: 55.7 ± 6.8%, 57.6 ± 10.1%, and 53.5 ± 8.8%, respectively). We also performed isokinetic measurements only at an angular velocity of 1.05 rad·s^−1^ (60°·s^−1^), and for tests performed at this angular velocity a reference value is close to 80% [78]. It is unclear what between-group differences would occur at higher angular velocities (e.g., 5.23 rad·s^−1^), since angular velocity affects the isokinetic strength profiles of professional athletes, including soccer players [71].

Overall, in the current study, a pattern was observed in isokinetic contractions, in which HRT seemed to elicit greater increases in Qcon and Hcon after the training intervention, compared to the PLY and CON groups. In practice, soccer players with better scores in the bilateral isokinetic test would have an advantage when performing various muscle burst functions (jumps, kicks, sprints) using them during the game. This justifies the application of individualized pre- and in-season hamstrings strength programs. Unfortunately, the lack of implementation of effective hamstring strength protocols within the complex soccer training is a common problem in professional soccer.

### 4.3. Jumping Height Performance

De Villarreal et al. [79] suggested in a meta-analytical study that the percentage range of improvements in vertical jump height after PLY training is of 4.7–15%. Considering the impact of a traditional RT, more recent advanced analyses by Silva et al. [13] showed that an average 24.4% improvement in 1-RM during squats results in an approximate 6.8% increase in CMJ, and a 22% increase in 1-RM results in a similar 6.7% average enhancement in SJ in high-level soccer players. With this in mind, but contrary to our hypothesis, it was interesting to note that the high-impact PLY protocol, which included various bilateral jump drills, was poorly effective in developing vertical jump performance. Specifically, soccer players in the PLY group did not benefit from the effects of explosive strength training on lower extremities when performing jump tests (CMJ and SJ). In all research groups, there were a significant pre- to post-training increases in CMJ and SJ heights performance (CMJ_height_: HRT = ∆5.9%; PLY = ∆3.3%; CG = ∆2.8%; SJ_height_: HRT = ∆7.2%; PLY = ∆3.5%; CG = ∆2.5%), but these improvements (in absolute and percentage terms) were two-fold greater with HRT than with the PLY protocol (especially in SJ_height_). It has been previously indicated that slow SSC jumps (i.e., CMJ) are likely to benefit more from PLY training than concentric jumps (i.e., SJ) [80]. However, we observed that the gain obtained with CMJ was not significantly different than that obtained with SJ in either HRT or PLY. Given the specificity of the type of contraction that is tracked during training, one would expect a greater positive effect of PLY training on CMJ than on SJ.

It is widely accepted that an athlete’s musculoskeletal system experiences extremely high impact forces during foot contact with the ground during high-intensity activity. In this study, multiple high-impact PLY exercises were performed as part of the PLY protocol (a total of 92-foot contacts/session in week 1, ending with 168-foot contacts/session in week 8). We speculated that muscle strength stimulation during each PLY session (e.g., DJs, single- and double-leg vertical and lateral hurdle jumps, horizontal and lateral bounding, power skipping) would be highly effective for developing explosive strength (power) of the lower extremities. Moreover, given that the effectiveness of PLY depends on plethora factors, such as intermuscular coordination, muscle power, and biomechanical specificity, it was somewhat surprising that the PLY group was not superior in vertical jump performance compared to HRT. In this context, heavy general lower-extremity exercise such as the squat is relatively more effective in developing intramuscular coordination, whereas loaded and/or high-impact jumps are more effective in developing intermuscular coordination. Interestingly, improvements in explosive actions are revealed earlier following the RT than the PLY programs [50].

Jumping height is a measure of performance that changes with alterations in 1-RM. Thus, the post-test scores and kinetics of CMJ_height_ and SJ_height_ observed in the HRT group were likely a result of maximal strength increases in the lower-extremity muscles (both quadriceps and calf muscles, inferred from CMJ and SJ tests, respectively). Resistance training focuses on the vertical component during triple extension of the lower body (i.e., ankle, knee, and hip), as in various squat-type exercises (e.g., full- or half-squat), as these are considered more similar to explosive-type activities (i.e., sprinting and jumping). However, we hypothesized that PLY-induced enhancements of explosive strength by improving motor unit synchronization, SSC efficiency, and/or muscle-tendon stiffness would elicit greater jumping performance in the PLY group. The increase in vertical jump scores observed in the HRT group may also explain the parallel increase in peak torques of knee flexor and extensor muscles in the current study (i.e., Qcon and Hcon), which were, as mentioned earlier, almost two-fold greater in the HRT group compared to the PLY group. Our results are consistent with related studies that have reported that Qcon and Hcon, assessed at slow speed and in concentric mode, showed correlations with SJ (*r* = 0.48; *p* < 0.001) [81]. Interestingly, another study showed higher correlation between Qcon isokinetic testing (at 180°·s^−1^ angular velocity) and vertical jumping height [39]. In addition, the CMJ and SJ test results in the HRT group may again have been due to excessive resistance loads (80–90% of 1-RM), as the 30–45% 1-RM range during a traditional squat is considered the optimal resistance for maximal power output in dynamic (ballistic) exercises/measurements, like jumping [82]. The mechanism is that lifting light or moderate loads provides the achievement of higher acceleration rates, which increase the contribution of this vector value to the total amount of applied force [82]. Based on this, it can be speculated that the improvement in vertical jump performance in the HRT group was even suboptimal, as they did not maximize mechanical power output. In the study by Brito et al. [12], low-volume RT induced improvements in jump performance, such as SJ (+10%, *p* < 0.05), and the five-jump test (+4%, *p* < 0.001). However, the training load within this RT protocol was insufficient to significantly improve CMJ scores. Chelly et al. [30] reported increases in CMJ and SJ height scores by 2.5% and 8.3%, respectively, after 8-week, bi-weekly PLY training program using body mass as resistance in 19-year-old male soccer players. Nevertheless, our results are consonant with those of de Villarreal et al. [65], who observed similar improvements in vertical jump performance after combined training protocol (i.e., using full-squat, parallel-squat, loaded CMJ and PLY exercises), HRT, and power-oriented strength training alone (i.e., using PLY or loaded CMJ training protocols). 

### 4.4. Limitations

There are some limitations to this study that need to be considered. The 8-week training intervention may not have revealed training adaptations that might have developed with longer programs (>10 weeks). In addition, this study did not include a combined HRT and PLY group, as has been done in some previous studies. A practical limitation is that due to daily variations in neuromuscular performance, the real 1-RM values for a given participant and exercise may change during a training intervention (from one training session to the next). Therefore, we cannot be sure that the workloads (1-RM percentage-based RT, (%1-RM)) used in any particular training session fully match those intended and produced a certain training effect. It would also be advisable to monitor the repetition velocity loss during each resistance set to get an objective indicator of the actual degree of fatigue. Moreover, it was impossible to control all variables related to total training load, as well as many other confounding factors (i.e., sleep, diet, and training-induced fatigue). Another limitation of this study was that we did not measure isokinetic peak torques at higher angular velocities (more than 60°·s^−1^) or in eccentric mode. We also did not measure morphological changes, such as quadriceps muscle cross-sectional area, or muscle pennation angle, which correlate well with maximal strength. Heavy-resistance training induces greater muscle fiber hypertrophy than other forms of physical training [83]. Finally, the study lacked a direct analysis of physiological and/or biochemical markers that could help elucidate the possible mechanisms behind training-induced neuromuscular functional improvements in the soccer players tested. Future training studies investigating these interesting issues are warranted to provide stronger and more in-depth evidence.

## 5. Conclusions

In conclusion, few short-term differences were observed between HRT and PLY protocols incorporated into a standard soccer training regime over the intervention period. These methods, among other benefits, are likely to provoke a transient enhancement in muscle contractile properties (i.e., PAP effect). The outcomes of this study suggest that pre-season HRT is comparable to or more effective than PLY and CG in improving measures of sprint performance in both the acceleration (0–10 m) and maximal-velocity (0–30 m) phases (percent progress was greater in PLY), 1-RM half-back squat (but only in relative terms when compared to PLY), peak isokinetic torque of the knee extensors and flexors (especially in Qcon), and vertical jump performance (CMJ and SJ tests). The latter was rather unexpected, since the high-impact PLY protocol (range of 96–172 foot contacts during the 8-week intervention) included many vertical-type unloaded jump exercises very similar to the biomechanical structure of specific neuromuscular tasks like CMJ and SJ. The study showed some transference effects, such as between strength gains (1-RM half-back squat, Qcon and Hcon) and sprint performance over short and longer distances. From a practical standpoint, optimization of RT protocols should also match the soccer-specific technical skills of soccer players. Irrespective of the research group, an increased risk of sports injury (especially injury to the biceps femoris muscle) was found in more than 70% of soccer players (H/Q ratio < 60%). Based on this, coaches, strength and conditioning specialists, and medical staff should identify individuals with knee muscle imbalances, expressed as high quadriceps strength and low H/Q ratio, and apply individualized pre- and in-season hamstrings strength programs. Future longitudinal studies should determine what physiological/biochemical adaptations are responsible for the observed neuromuscular functional changes. The final and general conclusion is that during the pre-competitive season, additional HRT and PLY drills/exercises as a substitute for standard soccer training (i.e., lower-limb resistive exercises) as part of a regular 90 min practice twice a week for 8 weeks, can produce physical performance-enhancing effects in professional soccer players.

## Figures and Tables

**Table 1:** Detailed characteristics of the research groups at the start of the intervention period.

Variable	HRT (*n* = 15)	PLY (*n* = 15)	CG (*n* = 17)
Age (years)	22.1 ± 3.15	22.5 ± 3.20	22.1 ± 4.21
Soccer experience (years)	13.2 ± 1.26	15.1 ± 1.88	14.4 ± 1.47
Body height (cm)	181.4 ± 5.47	180.4 ± 4.51	179.7 ± 6.31
Body mass (kg)	75.2 ± 6.16	73.7 ± 3.90	73.9 ± 5.51
Body mass index (kg·m^2^)	22.8 ± 1.47	22.7 ± 0.97	22.9 ± 1.53
Body fat (%)	12.2 ± 3.45	11.7 ± 3.04	13.8 ± 3.45
Fat-free mass (kg)	66.1 ± 6.38	63.3 ± 3.79	63.7 ± 4.84

Values are presented as mean ± SD; HRT: heavy-resistance group; PLY: plyometric group; CG: control group. There were no significant differences between groups and within groups before and after the 8-week period.

**Table 2:** Details of heavy-resistance training protocol *.

	Week 1	Week 2	Week 3	Week 4	Week 5	Week 6	Week 7	Week 8
	80% 1-RM	80% 1-RM	80% 1-RM	85% 1-RM	85% 1-RM	85% 1-RM	90% 1-RM	90% 1-RM
Exercises	Sets × Repetitions							
Half-back squat	3 × 10	3 × 10	3 × 10	3 × 8	3 × 8	3 × 8	3 × 6	3 × 6
Leg press	3 × 10	3 × 10	3 × 10	3 × 8	3 × 8	3 × 8	3 × 6	3 × 6
Leg extension	3 × 10	3 × 10	3 × 10	3 × 8	3 × 8	3 × 8	3 × 6	3 × 6
Leg curl	3 × 10	3 × 10	3 × 10	3 × 8	3 × 8	3 × 8	3 × 6	3 × 6
Lunges	3 × 10	3 × 10	3 × 10	3 × 8	3 × 8	3 × 8	3 × 6	3 × 6
Calf raises	3 × 10	3 × 10	3 × 10	3 × 8	3 × 8	3 × 8	3 × 6	3 × 6

* RM—repetition maximum.

**Table 3:** Details of plyometric training protocol.

Week	Exercise	Sets	Repetitions	Total Ground Contacts
1	Leg lifts	2	8	92
	Plank kicks	2	8	
	Leg flexes	2	15	
	Leg kicks	2	15	
2	Ankle hops	3	10	96
	High knees	3	10	
	Split jumps	3	6	
	Cone hops	3	6	
3	Skip hops	3	8	102
	Freezes	3	8	
	Pogo hopping	4	7	
	Box jumps	4	7	
4	Drop landings	3	10	108
	Vertical jumps in place	3	10	
	Horizontal jumps	3	8	
	Split squat drop lands	3	8	
5	Power skipping	3	12	120
	Unilateral pogo hops	3	12	
	Multiple horizontal rebounds	4	6	
	Alternate leg bounding	4	6	
6	Tuck jumps	4	8	128
	Counter jumps	4	8	
	Multiple bounding	4	8	
	Drop jumps	4	8	
7	Single-leg forward hop and stick	4	10	160
	Single-leg hurdle jump (35 cm)	4	10	
	Single-leg lateral cone jump (40 cm)	4	10	
	Power skipping	4	10	
8	Double-leg hurdle jump (90 cm)	6	7	168
	Double-leg depth jump (70 cm)	6	7	
	Double-leg lateral cone jump (40 cm)	6	7	
	Power skipping	6	7	

**Table 4:** Test–retest reliability of the performance tests.

Criterion Measures	ICC	α	CV%
10 m sprint	0.91	0.91	1.1
30 m sprint	0.89	0.89	1.3
1-RM	0.93	0.93	2.2
CMJ	0.95	0.95	2.5
SJ	0.93	0.93	2.1
Qcon	0.96	0.96	2.5
Hcon	0.95	0.95	2.3

ICC = Intraclass correlation coefficient; α = Cronbach’s alpha reliability coefficients; CV = Coefficient of variation; 1-RM = One repetition maximum half-back squat; CMJ = Countermovement jump; SJ = Squat jump; Qcon = Peak concentric isokinetic knee extensor torque; Hcon = Peak concentric isokinetic knee flexor torque.

**Table 5:** Number of athletes (in %) who presented H/Q ratio lower than 0.6.

Soccer Group	HRT (*n* = 15)	PLY (*n* = 15)	CG (*n* = 17)
H/Q ratio < 0.60	12 (80.0%)	10 (66.7%)	11 (64.7%)

H/Q ratio = knee hamstring/quadriceps strength ratio.

## Data Availability

The raw data supporting the conclusions of this article will be made available by the corresponding author upon reasonable request.

## References

[1] Coratella G., Beato M., Cè E., Scurati R., Milanese C., Schena F., Esposito F. (2019). Effects of in-season enhanced negative work-based vs traditional weight training on change of direction and hamstrings-to-quadriceps ratio in soccer players. Biol. Sport..

[2] Owen A., Dunlop G., Rouissi M., Chtara M., Paul D., Zouhal H., Wong D.P. (2015). The relationship between lower-limb strength and match-related muscle damage in elite level professional European soccer players. J. Sports Sci..

[3] Sánchez-Ureña B., Rojas-Valverde D., Gutiérrez-Vagas J.C., Gutiérrez-Vagas R., Mjaanes J.M. (2022). Lower limb’s lateral and functional asymmetries and mechanical performance in professional soccer players. Med. Sci. Sports Exerc..

[4] Falces-Prieto M., Saez de Villarreal-Saez E., Raya-González J., González-Fernández F.T., Clemente F.M., Badicu G., Murawska-Ciałowicz E. (2021). The Differentiate Effects of Resistance Training with or without External Load on Young Soccer Players’ Performance and Body Composition. Front. Physiol..

[5] Nuñez F.J., De Hoyo M., López A.M., Sañudo B., Otero-Esquina C., Sanchez H., Gonzalo-Skok O. (2019). Eccentric-concentric ratio: A key factor for defining strength training in soccer. Int. J. Sports Med..

[6] Makaruk H., Starzak M., Płaszewski M., Winchester J.B. (2022). Internal Validity in Resistance Training Research: A Systematic Review. J. Sports Sci. Med..

[7] Nishioka T., Okada J. (2022). Ballistic Exercise Versus Heavy Resistance Exercise Protocols: Which Resistance Priming Is More Effective for Improving Neuromuscular Performance on the Following Day?. J. Strength Cond. Res..

[8] Bauer P., Uebellacker F., Mitter B., Aigner A.J., Hasenoehrl T., Ristl R., Tschan H., Seitz L.B. (2019). Combining higher-load and lower-load resistance training exercises: A systematic review and meta-analysis of findings from complex training studies. J. Sci. Med. Sport..

[9] Cormier P., Freitas T.T., Seaman K. (2021). A systematic review of resistance training methodologies for the development of lower body concentric mean power, peak power, and mean propulsive power in team-sport athletes. Sports Biomech..

[10] Nevin J. (2019). Autoregulated resistance training: Does velocity-based training represent the future?. Strength Cond. J..

[11] Davies T., Orr R., Halkai M., Hackett D. (2016). Effect of training leading to repetition failure on muscular strength: A systematic review and meta-analysis. Sports Med..

[12] Brito J., Vasconcellos F., Oliveira J., Krustrup P., Rebelo A. (2014). Short-term performance effects of three different low-volume strength-training programmes in college male soccer players. J. Hum. Kinet..

[13] Silva J.R., Nassis G.P., Rebelo A. (2015). Strength training in soccer with a specific focus on highly trained players. Sports Med.-Open..

[14] Latella C., Teo W.P., Drinkwater E.J., Kendall K., Haff G.G. (2019). The acute neuromuscular responses to cluster set resistance training: A systematic review and meta-analysis. Sports Med..

[15] Loturco I., Pereira L.A., Reis V.P., Zanetti V., Bishop C., McGuigan M.R. (2022). Traditional free-weight vs. variable resistance training applied to elite young soccer players during a short preseason: Effects on strength, speed, and power performance. J. Strength Cond. Res..

[16] Katushabe E.T., Kramer M. (2020). Effects of combined power band resistance training on sprint speed, agility, vertical jump height, and strength in collegiate soccer players. Int. J. Exerc. Sci..

[17] Maio Alves J.M.V., Rebelo A.N., Abrantes C., Sampaio J. (2010). Short-Term Effects of Complex and Contrast Training in Soccer Players’ Vertical Jump, Sprint, and Agility Abilities. J. Strength Cond. Res..

[18] Kotzamanidis C., Chatzopoulos D., Michailidis C., Papaiakovou G., Patikas D. (2005). The effect of a combined high-intensity strength and speed training program on the running and jumping ability of soccer players. J. Strength Cond. Res..

[19] Denadai B.S., Oliveira F.B., Camarda S.R., Ribeiro L., Greco C.C. (2017). Effects of low-load resistance training with blood flow restriction on muscle size and strength of professional soccer players with muscle imbalance. Int. J. Appl. Exerc. Physiol..

[20] McBride J.M., Triplett-McBride T., Davie A., Newton R.U. (2002). The effect of heavy-vs. light-load jump squats on the development of strength, power, and speed. J. Strength Cond. Res..

[21] Ramirez-Campillo R., Sortwell A., Moran J., Afonso J., Clemente F.M., Lloyd R.S., Oliver J.L., Pedley J., Granacher U. (2023). Plyometric-Jump Training Effects on Physical Fitness and Sport-Specific Performance According to Maturity: A Systematic Review with Meta-analysis. Sports Med.-Open..

[22] van de Hoef P.A., Brauers J.J., van Smeden M., Backx F.J., Brink M.S. (2020). The effects of lower-extremity plyometric training on soccer-specific outcomes in adult male soccer players: A systematic review and meta-analysis. Int. J. Sports Physiol. Perform..

[23] Aloui G., Hermassi S., Hayes L.D., Bouhafs E.G., Chelly M.S., Schwesig R. (2021). Loaded Plyometrics and Short Sprints with Change-of-Direction Training Enhance Jumping, Sprinting, Agility, and Balance Performance of Male Soccer Players. Appl. Sci..

[24] Davies G., Riemann B.L., Manske R. (2015). Current concepts of plyometric exercise. Int. J. Sports Phys. Ther..

[25] de Hoyo M., Gonzalo-Skok O., Sañudo B., Carrascal C., Plaza-Armas J.R., Camacho-Candil F., Otero-Esquina C. (2016). Comparative effects of in-season full-back squat, resisted sprint training, and plyometric training on explosive performance in U-19 elite soccer players. J. Strength Cond. Res..

[26] Kobal R., Pereira L.A., Zanetti V., Ramirez-Campillo R., Loturco I. (2017). Effects of unloaded vs. loaded plyometrics on speed and power performance of elite young soccer players. Front. Physiol..

[27] Los Arcos A., Yanci J., Mendiguchia J., Salinero J.J., Brughelli M., Castagna C. (2014). Short-term training effects of vertically and horizontally oriented exercises on neuromuscular performance in professional soccer players. Int. J. Sports Physiol. Perform..

[28] Moran J., Ramirez-Campillo R., Liew B., Chaabene H., Behm D.G., García-Hermoso A., Izquierdo M., Granacher U. (2021). Effects of vertically and horizontally orientated plyometric training on physical performance: A meta-analytical comparison. Sports Med..

[29] Yanci J., Los Arcos A., Camara J., Castillo D., García A., Castagna C. (2016). Effects of horizontal plyometric training volume on soccer players’ performance. Res. Sports Med..

[30] Chelly M.S., Ghenem M.A., Abid K., Hermassi S., Tabka Z., Shephard R.J. (2010). Effects of in-season short-term plyometric training program on leg power, jump-and sprint performance of soccer players. J. Strength Cond. Res..

[31] Bogdanis G.C., Tsoukos A., Kaloheri O., Terzis G., Veligekas P., Brown L.E. (2019). Comparison between unilateral and bilateral plyometric training on single-and double-leg jumping performance and strength. J. Strength Cond. Res..

[32] Clemente F., Ramirez-Campillo R., Castillo D., Raya-González J., Rico-González M., Oliveira R., Rosemann T., Knechtle B. (2022). Effects of plyometric jump training on soccer player’s balance: A systematic review and meta-analysis of randomized-controlled trials. Biol. Sport..

[33] Manouras N., Papanikolaou Z., Karatrantou K., Kouvarakis P., Gerodimos V. (2016). The efficacy of vertical vs. horizontal plyometric training on speed, jumping performance and agility in soccer players. Int. J. Sports Sci. Coach..

[34] Perez-Gomez J., Olmedillas H., Delgado-Guerra S., Royo I.A., Vicente-Rodriguez G., Ortiz R.A., Chavarren J., Calbet J.A. (2008). Effects of weight lifting training combined with plyometric exercises on physical fitness, body composition, and knee extension velocity during kicking in football. Appl. Physiol. Nutr. Metab..

[35] Nakamura D., Suzuki T., Yasumatsu M., Akimoto T. (2012). Moderate running and plyometric training during off-season did not show a significant difference on soccer-related high-intensity performances compared with no-training controls. J. Strength Cond. Res..

[36] Ramirez-Campillo R., Moran J., Oliver J.L., Pedley J.S., Lloyd R.S., Granacher U. (2022). Programming plyometric-jump training in soccer: A review. Sports.

[37] de Villarreal E.S.S., González-Badillo J.J., Izquierdo M. (2007). Optimal warm-up stimuli of muscle activation to enhance short and long-term acute jumping performance. Eur. J. Appl. Physiol..

[38] Rodríguez-Rosell D., Torres-Torrelo J., Franco-Márquez F., González-Suárez J.M., González-Badillo J.J. (2017). Effects of light-load maximal lifting velocity weight training vs. combined weight training and plyometrics on sprint, vertical jump and strength performance in adult soccer players. J. Sci. Med. Sport..

[39] Requena B., González-Badillo J.J., de Villareal E.S.S., Ereline J., García I., Gapeyeva H., Pääsuke M. (2009). Functional performance, maximal strength, and power characteristics in isometric and dynamic actions of lower extremities in soccer players. J. Strength Cond. Res..

[40] Chelly M.S., Fathloun M., Cherif N., Amar M.B., Tabka Z., Van Praagh E. (2009). Effects of a back squat training program on leg power, jump, and sprint performances in junior soccer players. J. Strength Cond. Res..

[41] Boraczyński M., Boraczyński T., Podstawski R., Wójcik Z., Gronek P. (2020). Relationships between measures of functional and isometric lower body strength, aerobic capacity, anaerobic power, sprint and countermovement jump performance in professional soccer players. J. Hum. Kinet..

[42] McKinlay B.J., Wallace P., Dotan R., Long D., Tokuno C., Gabriel D.A., Falk B. (2018). Effects of plyometric and resistance training on muscle strength, explosiveness, and neuromuscular function in young adolescent soccer players. J. Strength Cond. Res..

[43] Hermassi S., Chelly M.S., Tabka Z., Shephard R.J., Chamari K. (2011). Effects of 8-week in-season upper and lower limb heavy resistance training on the peak power, throwing velocity, and sprint performance of elite male handball players. J. Strength Cond. Res..

[44] Impellizzeri F.M., Rampinini E., Coutts A.J., Sassi A.L.D.O., Marcora S.M. (2004). Use of RPE-based training load in soccer. Med. Sci. Sports Exerc..

[45] Ramírez-Campillo R., Andrade D.C., Izquierdo M. (2013). Effects of plyometric training volume and training surface on explosive strength. J. Strength Cond. Res..

[46] Castillo D., Raya-González J., Manuel Clemente F., Yanci J. (2020). The influence of youth soccer players’ sprint performance on the different sided games’ external load using GPS devices. Res Sports Med..

[47] Stølen T., Chamari K., Castagna C., Wisløff U. (2005). Physiology of soccer. Sports Med..

[48] Wyland T.P., Van Dorin J.D., Reyes G.F.C. (2015). Postactivation potentation effects from accommodating resistance combined with heavy back squats on short sprint performance. J. Strength Cond. Res..

[49] Loturco I., Kobal R., Gil S., Pivetti B., Kitamura K., Pereira L.A., Abad C.C., Nakamura F.Y. (2014). Differences in loaded and unloaded vertical jumping ability and sprinting performance between Brazilian elite under-20 and senior soccer players. Am. J. Sports Sci..

[50] Negra Y., Chaabene H., Sammoud S., Prieske O., Moran J., Ramirez-Campillo R., Nejmaoui A., Granacher U. (2020). The increased effectiveness of loaded versus unloaded plyometric jump training in improving muscle power, speed, change of direction, and kicking-distance performance in prepubertal male soccer players. Int. J. Sports Physiol. Perform..

[51] Faude O., Koch T., Meyer T. (2012). Straight sprinting is the most frequent action in goal situations in professional football. J. Sports Sci..

[52] Seitz L.B., Reyes A., Tran T.T., de Villarreal E.S., Haff G.G. (2014). Increases in lower-body strength transfer positively to sprint performance: A systematic review with meta-analysis. Sports Med..

[53] Comfort P., Stewart A., Bloom L., Clarkson B. (2014). Relationships between strength, sprint, and jump performance in well-trained youth soccer players. J. Strength Cond. Res..

[54] McBride J.M., Blow D., Kirby T.J., Haines T.L., Dayne A.M., Triplett N.T. (2009). Relationship between maximal squat strength and five, ten, and forty yard sprint times. J. Strength Cond. Res..

[55] Wisløff U., Castagna C., Helgerud J., Jones R., Hoff J. (2004). Strong correlation of maximal squat strength with sprint performance and vertical jump height in elite soccer players. Br. J. Sports Med..

[56] Styles W.J., Matthews M.J., Comfort P. (2016). Effects of strength training on squat and sprint performance in soccer players. J. Strength Cond. Res..

[57] Comfort P., Haigh A., Matthews M.J. (2012). Are changes in maximal squat strength during preseason training reflected in changes in sprint performance in rugby league players?. J. Strength Cond. Res..

[58] Chaouachi A., Brughelli M., Chamari K., Levin G.T., Ben Abdelkrim N., Laurencelle L., Castagna C. (2009). Lower limb maximal dynamic strength and agility determinants in elite basketball players. J. Strength Cond. Res..

[59] Lahti J., Huuhka T., Romero V., Bezodis I., Morin J.B., Häkkinen K. (2020). Changes in sprint performance and sagittal plane kinematics after heavy resisted sprint training in professional soccer players. PeerJ..

[60] Thomas K., Brownstein C., Dent J., Parker P., Goodall S., Howatson G. (2018). Neuromuscular fatigue and recovery after heavy resistance, jump, and sprint training. Med. Sci. Sports Exerc..

[61] Loturco I., Pereira L.A., Kobal R., Zanetti V., Kitamura K., Abad C.C.C., Nakamura F.Y. (2015). Transference effect of vertical and horizontal plyometrics on sprint performance of high-level U-20 soccer players. J. Sports Sci..

[62] Malisoux L., Francaux M., Nielens H., Theisen D. (2006). Stretch-shortening cycle exercises: An effective training paradigm to enhance power output of human single muscle fibers. J. Appl. Physiol..

[63] Herrero J.A., Izquierdo M., Maffiuletti N.A., Garcia-Lopez J. (2006). Electromyostimulation and plyometric training effects on jumping and sprint time. Int. J. Sports Med..

[64] Lloyd R.S., Radnor J.M., Croix M.B.D.S., Cronin J.B., Oliver J.L. (2016). Changes in sprint and jump performances after traditional, plyometric, and combined resistance training in male youth pre-and post-peak height velocity. J. Strength Cond. Res..

[65] de Villarreal E.S.S., Izquierdo M., Gonzalez-Badillo J.J. (2011). Enhancing jump performance after combined vs. maximal power, heavy-resistance, and plyometric training alone. J. Strength Cond. Res..

[66] Kobal R., Loturco I., Barroso R., Gil S., Cuniyochi R., Ugrinowitsch C., Roschel H., Tricoli V. (2017). Effects of different combinations of strength, power, and plyometric training on the physical performance of elite young soccer players. J. Strength Cond. Res..

[67] Markovic G., Mikulic P. (2010). Neuro-musculoskeletal and performance adaptations to lower-extremity plyometric training. Sports Med..

[68] Helgerud J., Kemi O.J., Hoff J., Hoff J., Helgerud J. (2003). Pre-season concurrent strength and endurance development in elite soccer players. Football (Soccer): New Developments in Physical Training Research.

[69] Hoff J., Helgerud J., Hoff J., Helgerud J. (2003). Maximal strength training enhances running economy and aerobic endurance performance. Football (Soccer): New Developments in Physical Training Research.

[70] Bogdanis G.C., Kalapotharakos V.I. (2015). Knee extension strength and hamstrings-to-quadriceps imbalances in elite soccer players. Int. J. Sports Med..

[71] Andrade M.D.S., De Lira C.A.B., Koffes F.D.C., Mascarin N.C., Benedito-Silva A.A., Da Silva A.C. (2012). Isokinetic hamstrings-to-quadriceps peak torque ratio: The influence of sport modality, gender, and angular velocity. J. Sports Sci..

[72] Coratella G., Bellin G., Beato M., Schena F. (2015). Fatigue affects peak joint torque angle in hamstrings but not in quadriceps. J. Sports Sci..

[73] Ruas C.V., Minozzo F., Pinto M.D., Brown L.E., Pinto R.S. (2015). Lower-extremity strength ratios of professional soccer players according to field position. J. Strength Cond. Res..

[74] Lutz F.D., Cleary C.J., Moffatt H.M., Sullivan V.E., LaRoche D.P., Cook S.B. (2022). Comparison of the H: Q Ratio Between the Dominant and Nondominant Legs of Soccer Players: A Meta-Analysis. Sports Health..

[75] Denadai B.S., de Oliveira F.B.D., Camarda S.R.D.A., Ribeiro L., Greco C.C. (2016). Hamstrings-to-quadriceps strength and size ratios of male professional soccer players with muscle imbalance. Clin. Physiol. Funct. Imaging..

[76] Croisier J.L., Ganteaume S., Binet J., Genty M., Ferret J.M. (2008). Strength imbalances and prevention of hamstring injury in professional soccer players: A prospective study. Am. J. Sports Med..

[77] de Lira C.A., Mascarin N.C., Vargas V.Z., Vancini R.L., Andrade M.S. (2017). Isokinetic knee muscle strength profile in Brazilian male soccer, futsal, and beach soccer players: A cross-sectional study. Int. J. Sports Phys. Ther..

[78] Baroni B.M., Ruas C.V., Ribeiro-Alvares J.B., Pinto R.S. (2020). Hamstring-to-quadriceps torque ratios of professional male soccer players: A systematic review. J. Strength Cond. Res..

[79] de Villarreal E.S.S., Kellis E., Kraemer W.J., Izquierdo M. (2009). Determining variables of plyometric training for improving vertical jump height performance: A meta-analysis. J. Strength Cond. Res..

[80] Markovic G. (2007). Does plyometric training improve vertical jump height? A meta-analytical review. Br. J. Sports Med..

[81] Lehance C., Binet J., Bury T., Croisier J.L. (2008). Muscular strength, functional performances and injury risk in professional and junior elite soccer players. Scand. J. Med. Sci. Sports.

[82] Loturco I., Nakamura F.Y., Kobal R., Gil S., Pivetti B., Pereira L.A., Roschel H. (2016). Traditional periodization versus optimum training load applied to soccer players: Effects on neuromuscular abilities. Int. J. Sports Med..

[83] Monserdà-Vilaró A., Balsalobre-Fernández C., Hoffman J.R., Alix-Fages C., Jiménez S.L. (2023). Effects of Concurrent Resistance and Endurance Training Using Continuous or Intermittent Protocols on Muscle Hypertrophy: Systematic Review With Meta-Analysis. J. Strength Cond. Res..

